# *catena*-Poly[sodium [[tris(3-methyl­pyridine-2-carboxylato)europate(III)]-μ-3-methylpyridine-2-carboxylato] trihydrate]

**DOI:** 10.1107/S160053681003151X

**Published:** 2010-08-18

**Authors:** Sung Kwon Kang

**Affiliations:** a Department of Chemistry, Chungnam National University, Daejeon 305-764, Republic of Korea

## Abstract

In the title structure, {Na[Eu(C_7_H_6_NO_2_)_4_]·3H_2_O}_*n*_, the Eu^III^ atom is nine-coordin­ated within a slightly distorted tricapped trigonal-prismatic coordination geometry defined by five carboxyl­ate-O atoms and four pyridine-N atoms. One of the carboxyl­ate ligands bridges the Eu cations, forming a one-dimensional coordination polymer along the *b* axis. The Eu—O bond distances lie within the range 2.362 (4)–2.461 (4) Å. In the crystal structure, inter­molecular O—H⋯O hydrogen bonds link the polymers into a three-dimensional network.

## Related literature

For general background to pyridine carb­oxy­lic complexes, see: Seo *et al.* (2010 ▶); Kukovec *et al.* (2009 ▶); Hong *et al.* (2008 ▶); Soares-Santos *et al.* (2006 ▶). For the syntheses and structures of Eu complexes, see: Lis *et al.* (2009 ▶); Godlewska *et al.* (2008 ▶); Legendziewicz *et al.* (2002 ▶).
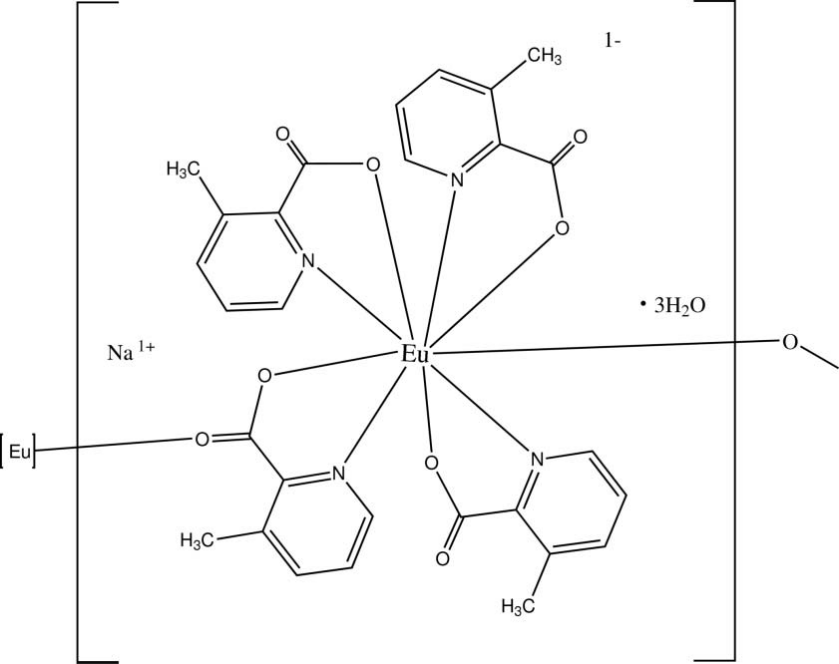


## Experimental

### 

#### Crystal data

Na[Eu(C_7_H_6_NO_2_)_4_]·3H_2_O
*M* *_r_* = 773.51
Monoclinic, 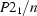

*a* = 11.721 (3) Å
*b* = 12.615 (4) Å
*c* = 21.133 (6) Å
β = 96.585 (7)°
*V* = 3104.1 (16) Å^3^
*Z* = 4
Mo *K*α radiation
μ = 2.10 mm^−1^
*T* = 233 K
0.22 × 0.15 × 0.14 mm

#### Data collection

Bruker SMART CCD area-detector diffractometer
Absorption correction: multi-scan (*SADABS*; Bruker, 2002 ▶) *T*_min_ = 0.583, *T*_max_ = 0.741
25592 measured reflections
5782 independent reflections
4104 reflections with *I* > 2σ(*I*)
*R*_int_ = 0.088

#### Refinement

*R*[*F* ^2^ > 2σ(*F* ^2^)] = 0.045
*wR*(*F* ^2^) = 0.148
*S* = 1.05
5782 reflections
428 parameters
6 restraints
H atoms treated by a mixture of independent and constrained refinement
Δρ_max_ = 1.77 e Å^−3^
Δρ_min_ = −1.63 e Å^−3^

### 

Data collection: *SMART* (Bruker, 2002 ▶); cell refinement: *SAINT* (Bruker, 2002 ▶); data reduction: *SAINT*; program(s) used to solve structure: *SHELXS97* (Sheldrick, 2008 ▶); program(s) used to refine structure: *SHELXL97* (Sheldrick, 2008 ▶); molecular graphics: *DIAMOND* (Brandenburg, 2010 ▶); software used to prepare material for publication: *WinGX* publication routines (Farrugia, 1999 ▶).

## Supplementary Material

Crystal structure: contains datablocks global, I. DOI: 10.1107/S160053681003151X/tk2695sup1.cif

Structure factors: contains datablocks I. DOI: 10.1107/S160053681003151X/tk2695Isup2.hkl

Additional supplementary materials:  crystallographic information; 3D view; checkCIF report

## Figures and Tables

**Table 1:** Selected bond lengths (Å)

Eu1—O28	2.362 (4)
Eu1—O18	2.366 (4)
Eu1—O8	2.375 (5)
Eu1—O38	2.445 (4)
Eu1—O39^i^	2.461 (4)
Eu1—N11	2.606 (5)
Eu1—N1	2.619 (6)
Eu1—N31	2.683 (6)
Eu1—N21	2.745 (6)

Symmetry code: (i) 
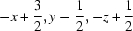
.

**Table 2:** Hydrogen-bond geometry (Å, °)

*D*—H⋯*A*	*D*—H	H⋯*A*	*D*⋯*A*	*D*—H⋯*A*
O41—H41*A*⋯O29^i^	0.82 (2)	2.08 (6)	2.767 (7)	141 (8)
O41—H41*B*⋯O19	0.81 (2)	2.02 (3)	2.809 (8)	164 (9)
O42—H42*A*⋯O9	0.81 (4)	2.41 (10)	2.774 (9)	108 (8)
O42—H42*B*⋯O43	0.82 (4)	2.08 (4)	2.852 (10)	158 (10)
O43—H43*A*⋯O41^ii^	0.82 (3)	2.02 (6)	2.769 (9)	152 (11)
O43—H43*B*⋯O29^i^	0.83 (2)	2.14 (6)	2.884 (8)	149 (10)

Symmetry codes: (i) 
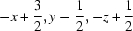
; (ii) 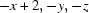.

**Fig. 1.**
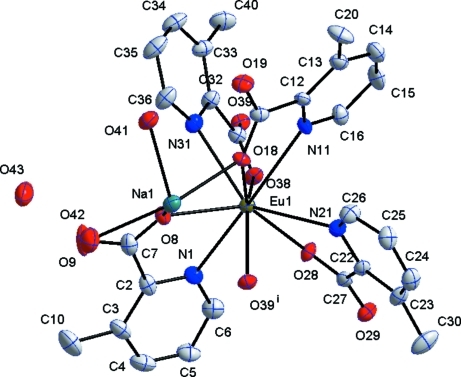
Molecular structure of the title complex showing the atom-numbering scheme and 50% probability ellipsoids. H atoms have been omitted for clarity. [Symmetry code: (i) -x + 3/2, y - 1/2, -z + 1/2]

**Fig. 2.**
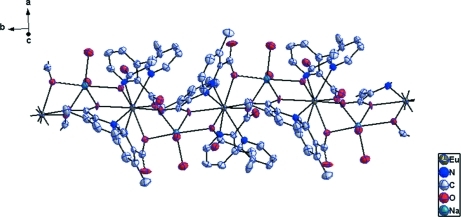
One-dimensional supramolecular chain along the b axis. H atoms have been omitted for clarity.

## supplementary crystallographic information

### Comment

Metal complexes of picolinic acid and their derivatives have been of considerable interest due to their adoption of various coordination modes and their interesting photophysical properties (Seo *et al.*, 2010; Kukovec *et al.*, 2009; Hong *et al.*, 2008; Soares-Santos *et al.*, 2006). Especially, Eu(III) complexes have been extensively studied due to their unique luminescence properties such as narrow emission bands and the excitation spectra of the five-dimensional_0_ — > 7F_0_ transition (Lis *et al.*, 2009; Godlewska *et al.*, 2008; Legendziewicz *et al.*, 2002). In this study, we report the synthesis and characterization of a Eu(III)-picolinic acid derivative in order to develop new lanthanide complexes for novel photoluminescent applications.

In the title one-dimensional coordination polymer, {Na[Eu(C_7_H_6_NO_2_)_4_] 3H_2_O}_n_, the Eu atom is nine-coordinate within a slightly distorted tricapped trigonal prismatic coordination geometry. The Eu(III) atom is coordinated to the five carboxylate-O atoms and four pyridine-N atoms. The Eu—O bond distances are within the range of 2.362 (4) - 2.461 (4) Å, Table 1, which are significantly shorter than the sum of the covalent radii of Eu and O atoms (2.66 Å). The dihedral angles between the pyridine rings and the carboxylate groups are in the range of 5.0 (4) - 24.7 (4) °. One of the carboxylate ligands bridges Eu cations to form a one-dimensional coordination polymer along the crystallographic *b* axis (Fig. 2). Intramolecular O—H···O hydrogen bonds link the uncoordinated water molecules to the coordinated water molecules, Table 2. In the crystal structure, intermolecular O—H···O hydrogen bonds link the polymers into a three-dimensional network, Table 2. The title compound exhibits an intense emission at 618 nm upon 396 nm excitation in PL spectra with 325 nm of He—Cd laser excitation wavelength.

### Experimental

Europium trichloride solution was prepared by dissolving EuCl_3_ 6H_2_O (0.37 g, 1.0 mmol; Aldrich) in absolute ethanol (20 ml) at room temperature with stirring. The ligand solution was prepared by dissolving 3-methylpicolinic acid (0.55 g, 4.0 mmol; Aldrich) in absolute ethanol (30 ml) at room temperature. The pH of the ligand solution was adjusted to about 6 with 2 N NaOH solution. The Eu solution was added drop wise and slowly to the ligand solution. The reaction mixture was stirred for 2 h at room temperature. Colourless crystals of (I) were obtained at room temperature over a period of a few weeks. The complex was recrystallized from distilled water.

### Refinement

The water-H atoms were located in a difference Fourier map and refined with O—H = 0.82±0.01 Å. The remaining H atoms were positioned geometrically and refined using a riding model with C—H = 0.94 - 0.97 Å, and with *U*_iso_(H) = 1.2*U*_eq_ (C) for aromatic-H and 1.5*U*_eq_(C) for methyl-H atoms. The maximum and minimum residual electron density peaks of 1.77 and -1.63 eÅ^-3^, respectively, were located 1.55 Å and 0.79 Å from the O39 and Eu1 atoms, respectively.

### Crystal data

**Table:**

Na[Eu(C_7_H_6_NO_2_)_4_]·3H_2_O	*F*(000) = 1552
*M**_r_* = 773.51	*D*_x_ = 1.655 Mg m^−^^3^
Monoclinic, *P*2_1_/*n*	Mo *K*α radiation, λ = 0.71073 Å
Hall symbol: -P 2yn	Cell parameters from 3070 reflections
*a* = 11.721 (3) Å	θ = 2.5–20.6°
*b* = 12.615 (4) Å	µ = 2.10 mm^−^^1^
*c* = 21.133 (6) Å	*T* = 233 K
β = 96.585 (7)°	Block, colourless
*V* = 3104.1 (16) Å^3^	0.22 × 0.15 × 0.14 mm
*Z* = 4	

### Data collection

**Table:**

Bruker SMART CCD area-detector diffractometer	4104 reflections with *I* > 2σ(*I*)
φ and ω scans	*R*_int_ = 0.088
Absorption correction: multi-scan (*SADABS*; Bruker, 2002)	θ_max_ = 25.5°, θ_min_ = 1.9°
*T*_min_ = 0.583, *T*_max_ = 0.741	*h* = −14→14
25592 measured reflections	*k* = −15→15
5782 independent reflections	*l* = −25→25

### Refinement

**Table:**

Refinement on *F*^2^	6 restraints
Least-squares matrix: full	H atoms treated by a mixture of independent and constrained refinement
*R*[*F*^2^ > 2σ(*F*^2^)] = 0.045	*w* = 1/[σ^2^(*F*_o_^2^) + 1.5543*P*] where *P* = (*F*_o_^2^ + 2*F*_c_^2^)/3
*wR*(*F*^2^) = 0.148	(Δ/σ)_max_ = 0.001
*S* = 1.05	Δρ_max_ = 1.77 e Å^−^^3^
5782 reflections	Δρ_min_ = −1.63 e Å^−^^3^
428 parameters	

### Special details

**Table:**

Geometry. All e.s.d.'s (except the e.s.d. in the dihedral angle between two l.s. planes) are estimated using the full covariance matrix. The cell e.s.d.'s are taken into account individually in the estimation of e.s.d.'s in distances, angles and torsion angles; correlations between e.s.d.'s in cell parameters are only used when they are defined by crystal symmetry. An approximate (isotropic) treatment of cell e.s.d.'s is used for estimating e.s.d.'s involving l.s. planes.

### Fractional atomic coordinates and isotropic or equivalent isotropic displacement parameters (Å^2^)

**Table:**

	*x*	*y*	*z*	*U*_iso_*/*U*_eq_	
Eu1	0.73313 (3)	0.28658 (2)	0.240306 (15)	0.02324 (14)	
N1	0.9295 (5)	0.3407 (4)	0.3039 (3)	0.0301 (13)	
C2	1.0234 (6)	0.2942 (5)	0.2843 (4)	0.0314 (17)	
C3	1.1302 (7)	0.2917 (6)	0.3230 (4)	0.043 (2)	
C4	1.1340 (8)	0.3421 (7)	0.3822 (4)	0.053 (2)	
H4	1.2033	0.3425	0.4095	0.063*	
C5	1.0396 (7)	0.3910 (6)	0.4014 (4)	0.044 (2)	
H5	1.0435	0.4255	0.441	0.053*	
C6	0.9388 (7)	0.3882 (6)	0.3610 (4)	0.0413 (19)	
H6	0.8735	0.4212	0.3741	0.05*	
C7	1.0039 (7)	0.2447 (6)	0.2190 (4)	0.0374 (18)	
O8	0.9005 (4)	0.2233 (3)	0.1990 (2)	0.0311 (11)	
O9	1.0864 (5)	0.2306 (5)	0.1881 (3)	0.067 (2)	
C10	1.2366 (8)	0.2388 (8)	0.3051 (6)	0.069 (3)	
H10A	1.2968	0.2429	0.3405	0.104*	
H10B	1.2615	0.2743	0.2683	0.104*	
H10C	1.2201	0.165	0.2947	0.104*	
N11	0.5296 (5)	0.3202 (5)	0.1801 (3)	0.0291 (13)	
C12	0.4908 (6)	0.2464 (5)	0.1358 (3)	0.0268 (15)	
C13	0.3748 (7)	0.2430 (6)	0.1098 (4)	0.0355 (17)	
C14	0.3025 (7)	0.3207 (6)	0.1293 (4)	0.043 (2)	
H14	0.2244	0.32	0.1133	0.052*	
C15	0.3431 (7)	0.3994 (7)	0.1720 (4)	0.051 (2)	
H15	0.2951	0.4541	0.1836	0.061*	
C16	0.4579 (6)	0.3936 (6)	0.1967 (4)	0.0381 (18)	
H16	0.4863	0.4446	0.2269	0.046*	
C17	0.5823 (6)	0.1697 (5)	0.1196 (3)	0.0312 (16)	
O18	0.6691 (4)	0.1589 (3)	0.1622 (2)	0.0276 (10)	
O19	0.5705 (5)	0.1243 (4)	0.0678 (2)	0.0409 (13)	
C20	0.3256 (7)	0.1569 (6)	0.0649 (4)	0.052 (2)	
H20A	0.3226	0.0908	0.0881	0.078*	
H20B	0.374	0.1481	0.0309	0.078*	
H20C	0.2487	0.1766	0.0468	0.078*	
N21	0.5686 (5)	0.1796 (5)	0.2975 (3)	0.0313 (14)	
C22	0.5719 (6)	0.2005 (5)	0.3608 (3)	0.0264 (15)	
C23	0.5236 (7)	0.1327 (5)	0.4029 (3)	0.0357 (17)	
C24	0.4677 (7)	0.0435 (6)	0.3758 (4)	0.0419 (19)	
H24	0.4342	−0.0046	0.4022	0.05*	
C25	0.4604 (7)	0.0241 (6)	0.3116 (4)	0.0416 (19)	
H25	0.4195	−0.0348	0.2936	0.05*	
C26	0.5146 (7)	0.0935 (6)	0.2739 (4)	0.0402 (19)	
H26	0.5131	0.079	0.2302	0.048*	
C27	0.6349 (6)	0.3030 (5)	0.3811 (3)	0.0296 (16)	
O28	0.6793 (4)	0.3518 (3)	0.3374 (2)	0.0294 (11)	
O29	0.6355 (5)	0.3339 (4)	0.4368 (2)	0.0386 (12)	
C30	0.5320 (9)	0.1480 (7)	0.4743 (4)	0.060 (3)	
H30A	0.6116	0.1599	0.491	0.09*	
H30B	0.5037	0.0851	0.4938	0.09*	
H30C	0.4862	0.2088	0.4838	0.09*	
N31	0.7520 (5)	0.3872 (4)	0.1301 (3)	0.0317 (14)	
C32	0.7060 (6)	0.4861 (5)	0.1258 (3)	0.0314 (16)	
C33	0.6665 (7)	0.5319 (5)	0.0673 (3)	0.0371 (18)	
C34	0.6810 (8)	0.4736 (6)	0.0131 (4)	0.048 (2)	
H34	0.6543	0.5012	−0.0273	0.058*	
C35	0.7342 (8)	0.3756 (6)	0.0175 (4)	0.051 (2)	
H35	0.7467	0.338	−0.0195	0.061*	
C36	0.7687 (7)	0.3338 (6)	0.0771 (4)	0.0407 (19)	
H36	0.8043	0.267	0.0804	0.049*	
C37	0.7039 (6)	0.5389 (5)	0.1895 (3)	0.0284 (15)	
O38	0.7132 (4)	0.4795 (3)	0.2383 (2)	0.0270 (11)	
O39	0.6971 (4)	0.6366 (3)	0.1914 (2)	0.0302 (11)	
C40	0.6091 (8)	0.6392 (6)	0.0615 (4)	0.052 (2)	
H40A	0.5529	0.6441	0.0917	0.079*	
H40B	0.571	0.6481	0.0186	0.079*	
H40C	0.6664	0.6942	0.0705	0.079*	
Na1	0.8432 (2)	0.0484 (2)	0.16277 (12)	0.0320 (6)	
O41	0.7863 (5)	0.0404 (4)	0.0495 (3)	0.0408 (13)	
H41A	0.777 (7)	−0.022 (2)	0.058 (4)	0.049*	
H41B	0.720 (3)	0.060 (6)	0.048 (4)	0.049*	
O42	1.0436 (6)	0.0189 (5)	0.1588 (3)	0.0574 (16)	
H42A	1.020 (9)	0.068 (5)	0.136 (4)	0.069*	
H42B	1.073 (8)	−0.003 (8)	0.128 (3)	0.069*	
O43	1.0969 (6)	−0.1006 (6)	0.0514 (3)	0.0682 (19)	
H43A	1.111 (9)	−0.090 (8)	0.015 (2)	0.082*	
H43B	1.028 (3)	−0.113 (8)	0.040 (5)	0.082*	

### Atomic displacement parameters (Å^2^)

**Table:**

	*U*^11^	*U*^22^	*U*^33^	*U*^12^	*U*^13^	*U*^23^
Eu1	0.0261 (2)	0.0200 (2)	0.0234 (2)	0.00011 (13)	0.00207 (15)	−0.00091 (13)
N1	0.032 (3)	0.023 (3)	0.035 (3)	−0.004 (3)	0.000 (3)	−0.005 (3)
C2	0.027 (4)	0.016 (3)	0.052 (5)	−0.004 (3)	0.006 (4)	0.001 (3)
C3	0.032 (4)	0.036 (4)	0.059 (6)	0.001 (3)	−0.007 (4)	0.008 (4)
C4	0.050 (6)	0.052 (5)	0.050 (5)	−0.002 (4)	−0.018 (4)	0.006 (4)
C5	0.047 (5)	0.041 (4)	0.041 (5)	−0.008 (4)	−0.009 (4)	−0.005 (4)
C6	0.048 (5)	0.030 (4)	0.045 (5)	−0.002 (3)	0.002 (4)	−0.003 (3)
C7	0.035 (5)	0.029 (4)	0.050 (5)	0.001 (3)	0.012 (4)	−0.001 (3)
O8	0.025 (3)	0.032 (3)	0.036 (3)	0.001 (2)	0.005 (2)	−0.005 (2)
O9	0.032 (3)	0.095 (5)	0.079 (5)	−0.023 (3)	0.030 (3)	−0.039 (4)
C10	0.040 (6)	0.073 (6)	0.093 (8)	0.014 (5)	−0.002 (6)	0.002 (6)
N11	0.020 (3)	0.035 (3)	0.033 (3)	0.005 (3)	0.002 (3)	−0.006 (3)
C12	0.025 (4)	0.029 (3)	0.026 (4)	0.003 (3)	−0.002 (3)	0.006 (3)
C13	0.034 (4)	0.037 (4)	0.035 (4)	0.001 (3)	−0.001 (4)	0.009 (3)
C14	0.033 (4)	0.043 (4)	0.054 (5)	0.007 (4)	0.003 (4)	0.005 (4)
C15	0.033 (5)	0.052 (5)	0.068 (6)	0.016 (4)	0.013 (4)	−0.004 (4)
C16	0.032 (4)	0.037 (4)	0.047 (5)	−0.003 (3)	0.010 (4)	0.002 (3)
C17	0.035 (4)	0.029 (4)	0.029 (4)	−0.003 (3)	−0.001 (3)	0.000 (3)
O18	0.025 (2)	0.026 (2)	0.029 (3)	−0.002 (2)	−0.005 (2)	−0.002 (2)
O19	0.048 (3)	0.040 (3)	0.032 (3)	−0.001 (2)	−0.002 (3)	−0.011 (2)
C20	0.043 (5)	0.048 (5)	0.059 (6)	0.000 (4)	−0.022 (4)	−0.005 (4)
N21	0.033 (3)	0.028 (3)	0.033 (3)	0.001 (3)	0.006 (3)	0.003 (3)
C22	0.030 (4)	0.021 (3)	0.028 (4)	0.001 (3)	0.005 (3)	0.007 (3)
C23	0.049 (5)	0.024 (4)	0.036 (4)	0.001 (3)	0.014 (4)	0.007 (3)
C24	0.042 (5)	0.031 (4)	0.054 (5)	−0.005 (3)	0.013 (4)	0.007 (4)
C25	0.039 (5)	0.034 (4)	0.053 (5)	−0.010 (3)	0.010 (4)	−0.006 (4)
C26	0.045 (5)	0.040 (4)	0.035 (4)	−0.009 (4)	0.003 (4)	−0.006 (3)
C27	0.030 (4)	0.033 (4)	0.025 (4)	0.008 (3)	0.003 (3)	0.001 (3)
O28	0.040 (3)	0.024 (2)	0.027 (2)	−0.002 (2)	0.015 (2)	−0.006 (2)
O29	0.049 (3)	0.040 (3)	0.027 (3)	−0.007 (3)	0.008 (2)	−0.001 (2)
C30	0.100 (8)	0.041 (5)	0.041 (5)	−0.026 (5)	0.018 (5)	0.005 (4)
N31	0.038 (4)	0.026 (3)	0.032 (3)	−0.004 (3)	0.007 (3)	−0.001 (2)
C32	0.039 (4)	0.023 (4)	0.033 (4)	−0.001 (3)	0.009 (3)	0.000 (3)
C33	0.045 (5)	0.035 (4)	0.031 (4)	−0.012 (3)	−0.002 (4)	0.005 (3)
C34	0.075 (6)	0.045 (5)	0.026 (4)	−0.016 (4)	0.005 (4)	0.001 (4)
C35	0.085 (7)	0.039 (5)	0.032 (4)	−0.011 (4)	0.019 (5)	−0.005 (4)
C36	0.062 (5)	0.027 (4)	0.037 (4)	−0.003 (4)	0.018 (4)	−0.003 (3)
C37	0.029 (4)	0.022 (4)	0.034 (4)	−0.003 (3)	0.000 (3)	0.001 (3)
O38	0.044 (3)	0.007 (2)	0.031 (3)	0.0076 (18)	0.009 (2)	0.0021 (18)
O39	0.039 (3)	0.022 (2)	0.029 (3)	−0.002 (2)	0.003 (2)	−0.006 (2)
C40	0.065 (6)	0.040 (5)	0.050 (5)	−0.005 (4)	−0.005 (5)	0.011 (4)
Na1	0.0388 (16)	0.0279 (14)	0.0295 (14)	0.0015 (12)	0.0048 (13)	−0.0019 (11)
O41	0.054 (4)	0.038 (3)	0.032 (3)	0.007 (3)	0.013 (3)	0.002 (2)
O42	0.057 (4)	0.058 (4)	0.061 (4)	0.007 (3)	0.020 (4)	−0.005 (3)
O43	0.049 (4)	0.107 (5)	0.052 (4)	−0.013 (4)	0.018 (4)	−0.001 (4)

### Geometric parameters (Å, °)

**Table:**

Eu1—O28	2.362 (4)	N21—C22	1.360 (9)
Eu1—O18	2.366 (4)	C22—C23	1.399 (9)
Eu1—O8	2.375 (5)	C22—C27	1.526 (9)
Eu1—O38	2.445 (4)	C23—C24	1.392 (10)
Eu1—O39^i^	2.461 (4)	C23—C30	1.513 (11)
Eu1—N11	2.606 (5)	C24—C25	1.371 (11)
Eu1—N1	2.619 (6)	C24—H24	0.94
Eu1—N31	2.683 (6)	C25—C26	1.386 (10)
Eu1—N21	2.745 (6)	C25—H25	0.94
N1—C6	1.341 (9)	C26—H26	0.94
N1—C2	1.354 (9)	C27—O29	1.238 (8)
C2—C3	1.415 (11)	C27—O28	1.271 (8)
C2—C7	1.507 (11)	O28—Na1^ii^	2.495 (5)
C3—C4	1.399 (12)	C30—H30A	0.97
C3—C10	1.501 (12)	C30—H30B	0.97
C4—C5	1.368 (12)	C30—H30C	0.97
C4—H4	0.94	N31—C36	1.342 (9)
C5—C6	1.376 (11)	N31—C32	1.359 (8)
C5—H5	0.94	C32—C33	1.395 (10)
C6—H6	0.94	C32—C37	1.505 (9)
C7—O9	1.242 (9)	C33—C34	1.389 (10)
C7—O8	1.266 (9)	C33—C40	1.510 (11)
O8—Na1	2.405 (5)	C34—C35	1.382 (12)
C10—H10A	0.97	C34—H34	0.94
C10—H10B	0.97	C35—C36	1.382 (11)
C10—H10C	0.97	C35—H35	0.94
N11—C16	1.325 (9)	C36—H36	0.94
N11—C12	1.360 (9)	C37—O39	1.236 (7)
C12—C13	1.408 (10)	C37—O38	1.269 (8)
C12—C17	1.512 (10)	O38—Na1^ii^	2.425 (5)
C13—C14	1.389 (11)	O39—Eu1^ii^	2.461 (4)
C13—C20	1.513 (11)	C40—H40A	0.97
C14—C15	1.388 (12)	C40—H40B	0.97
C14—H14	0.94	C40—H40C	0.97
C15—C16	1.389 (11)	Na1—O42	2.390 (7)
C15—H15	0.94	Na1—O41	2.412 (6)
C16—H16	0.94	Na1—O38^i^	2.425 (5)
C17—O19	1.229 (8)	Na1—O28^i^	2.495 (5)
C17—O18	1.287 (8)	O41—H41A	0.82 (2)
O18—Na1	2.470 (5)	O41—H41B	0.81 (2)
C20—H20A	0.97	O42—H42A	0.81 (4)
C20—H20B	0.97	O42—H42B	0.82 (4)
C20—H20C	0.97	O43—H43A	0.82 (3)
N21—C26	1.326 (9)	O43—H43B	0.83 (2)
			
O28—Eu1—O18	138.38 (16)	H20A—C20—H20C	109.5
O28—Eu1—O8	138.64 (17)	H20B—C20—H20C	109.5
O18—Eu1—O8	73.99 (16)	C26—N21—C22	119.2 (6)
O28—Eu1—O38	68.54 (14)	C26—N21—Eu1	124.5 (5)
O18—Eu1—O38	130.08 (15)	C22—N21—Eu1	113.3 (4)
O8—Eu1—O38	114.16 (15)	N21—C22—C23	122.6 (6)
O28—Eu1—O39^i^	82.10 (15)	N21—C22—C27	113.5 (6)
O18—Eu1—O39^i^	86.72 (15)	C23—C22—C27	123.9 (6)
O8—Eu1—O39^i^	73.99 (16)	C24—C23—C22	116.0 (7)
O38—Eu1—O39^i^	143.12 (15)	C24—C23—C30	119.1 (7)
O28—Eu1—N11	92.68 (17)	C22—C23—C30	124.8 (7)
O18—Eu1—N11	64.57 (16)	C25—C24—C23	121.6 (7)
O8—Eu1—N11	128.55 (17)	C25—C24—H24	119.2
O38—Eu1—N11	75.50 (17)	C23—C24—H24	119.2
O39^i^—Eu1—N11	129.43 (17)	C24—C25—C26	118.3 (7)
O28—Eu1—N1	76.71 (17)	C24—C25—H25	120.8
O18—Eu1—N1	136.50 (16)	C26—C25—H25	120.8
O8—Eu1—N1	64.04 (17)	N21—C26—C25	122.2 (7)
O38—Eu1—N1	79.96 (16)	N21—C26—H26	118.9
O39^i^—Eu1—N1	71.71 (16)	C25—C26—H26	118.9
N11—Eu1—N1	155.41 (18)	O29—C27—O28	125.5 (7)
O28—Eu1—N31	130.23 (16)	O29—C27—C22	118.9 (6)
O18—Eu1—N31	76.59 (16)	O28—C27—C22	115.5 (6)
O8—Eu1—N31	71.71 (17)	C27—O28—Eu1	129.5 (4)
O38—Eu1—N31	62.05 (15)	C27—O28—Na1^ii^	115.5 (4)
O39^i^—Eu1—N31	144.81 (17)	Eu1—O28—Na1^ii^	112.56 (18)
N11—Eu1—N31	70.19 (18)	C23—C30—H30A	109.5
N1—Eu1—N31	99.62 (18)	C23—C30—H30B	109.5
O28—Eu1—N21	61.14 (16)	H30A—C30—H30B	109.5
O18—Eu1—N21	77.87 (17)	C23—C30—H30C	109.5
O8—Eu1—N21	130.42 (17)	H30A—C30—H30C	109.5
O38—Eu1—N21	115.25 (16)	H30B—C30—H30C	109.5
O39^i^—Eu1—N21	64.34 (17)	C36—N31—C32	120.0 (6)
N11—Eu1—N21	69.23 (18)	C36—N31—Eu1	121.5 (4)
N1—Eu1—N21	121.48 (18)	C32—N31—Eu1	114.9 (4)
N31—Eu1—N21	138.30 (18)	N31—C32—C33	121.9 (6)
O28—Eu1—Na1	143.49 (12)	N31—C32—C37	113.2 (6)
O18—Eu1—Na1	40.69 (11)	C33—C32—C37	124.9 (6)
O8—Eu1—Na1	39.15 (12)	C34—C33—C32	116.9 (7)
O38—Eu1—Na1	146.40 (11)	C34—C33—C40	120.2 (7)
O39^i^—Eu1—Na1	61.90 (11)	C32—C33—C40	122.8 (7)
N11—Eu1—Na1	105.07 (13)	C35—C34—C33	120.9 (7)
N1—Eu1—Na1	96.20 (13)	C35—C34—H34	119.5
N31—Eu1—Na1	86.14 (13)	C33—C34—H34	119.5
N21—Eu1—Na1	95.37 (13)	C36—C35—C34	119.1 (7)
O28—Eu1—Na1^ii^	34.76 (11)	C36—C35—H35	120.5
O18—Eu1—Na1^ii^	147.06 (11)	C34—C35—H35	120.5
O8—Eu1—Na1^ii^	134.53 (12)	N31—C36—C35	120.9 (7)
O38—Eu1—Na1^ii^	33.77 (11)	N31—C36—H36	119.5
O39^i^—Eu1—Na1^ii^	114.16 (11)	C35—C36—H36	119.5
N11—Eu1—Na1^ii^	82.61 (13)	O39—C37—O38	124.2 (6)
N1—Eu1—Na1^ii^	76.06 (13)	O39—C37—C32	118.5 (6)
N31—Eu1—Na1^ii^	95.64 (12)	O38—C37—C32	117.2 (5)
N21—Eu1—Na1^ii^	88.33 (13)	C37—O38—Na1^ii^	118.8 (4)
Na1—Eu1—Na1^ii^	172.242 (13)	C37—O38—Eu1	126.9 (4)
C6—N1—C2	119.0 (6)	Na1^ii^—O38—Eu1	112.13 (17)
C6—N1—Eu1	123.7 (5)	C37—O39—Eu1^ii^	140.1 (4)
C2—N1—Eu1	115.3 (4)	C33—C40—H40A	109.5
N1—C2—C3	121.9 (7)	C33—C40—H40B	109.5
N1—C2—C7	114.6 (6)	H40A—C40—H40B	109.5
C3—C2—C7	123.4 (7)	C33—C40—H40C	109.5
C4—C3—C2	116.3 (8)	H40A—C40—H40C	109.5
C4—C3—C10	119.1 (8)	H40B—C40—H40C	109.5
C2—C3—C10	124.7 (8)	O42—Na1—O8	85.1 (2)
C5—C4—C3	121.7 (8)	O42—Na1—O41	96.8 (2)
C5—C4—H4	119.2	O8—Na1—O41	112.9 (2)
C3—C4—H4	119.2	O42—Na1—O38^i^	109.9 (2)
C4—C5—C6	118.1 (8)	O8—Na1—O38^i^	98.53 (18)
C4—C5—H5	120.9	O41—Na1—O38^i^	140.2 (2)
C6—C5—H5	120.9	O42—Na1—O18	154.5 (2)
N1—C6—C5	123.0 (8)	O8—Na1—O18	71.62 (17)
N1—C6—H6	118.5	O41—Na1—O18	83.39 (18)
C5—C6—H6	118.5	O38^i^—Na1—O18	84.22 (17)
O9—C7—O8	124.5 (8)	O42—Na1—O28^i^	87.1 (2)
O9—C7—C2	119.8 (7)	O8—Na1—O28^i^	159.7 (2)
O8—C7—C2	115.7 (6)	O41—Na1—O28^i^	86.55 (18)
C7—O8—Eu1	127.2 (5)	O38^i^—Na1—O28^i^	66.77 (15)
C7—O8—Na1	121.6 (4)	O18—Na1—O28^i^	118.29 (18)
Eu1—O8—Na1	102.29 (19)	O42—Na1—Eu1	122.20 (18)
C3—C10—H10A	109.5	O8—Na1—Eu1	38.56 (12)
C3—C10—H10B	109.5	O41—Na1—Eu1	113.60 (15)
H10A—C10—H10B	109.5	O38^i^—Na1—Eu1	76.54 (12)
C3—C10—H10C	109.5	O18—Na1—Eu1	38.65 (11)
H10A—C10—H10C	109.5	O28^i^—Na1—Eu1	139.58 (14)
H10B—C10—H10C	109.5	O42—Na1—Eu1^i^	99.58 (18)
C16—N11—C12	119.2 (6)	O8—Na1—Eu1^i^	131.19 (15)
C16—N11—Eu1	123.9 (5)	O41—Na1—Eu1^i^	114.62 (15)
C12—N11—Eu1	116.1 (4)	O38^i^—Na1—Eu1^i^	34.09 (10)
N11—C12—C13	121.4 (6)	O18—Na1—Eu1^i^	103.45 (13)
N11—C12—C17	113.8 (6)	O28^i^—Na1—Eu1^i^	32.67 (10)
C13—C12—C17	124.8 (6)	Eu1—Na1—Eu1^i^	109.18 (7)
C14—C13—C12	117.2 (7)	O42—Na1—H41A	97 (2)
C14—C13—C20	119.5 (7)	O8—Na1—H41A	132.3 (6)
C12—C13—C20	123.3 (7)	O41—Na1—H41A	19.4 (6)
C15—C14—C13	121.5 (8)	O38^i^—Na1—H41A	124.4 (12)
C15—C14—H14	119.2	O18—Na1—H41A	91.5 (18)
C13—C14—H14	119.2	O28^i^—Na1—H41A	67.2 (7)
C14—C15—C16	116.8 (7)	Eu1—Na1—H41A	127.3 (16)
C14—C15—H15	121.6	Eu1^i^—Na1—H41A	95.6 (7)
C16—C15—H15	121.6	O42—Na1—H42A	19.9 (10)
N11—C16—C15	123.7 (7)	O8—Na1—H42A	75 (2)
N11—C16—H16	118.2	O41—Na1—H42A	85 (2)
C15—C16—H16	118.2	O38^i^—Na1—H42A	127.8 (14)
O19—C17—O18	125.0 (7)	O18—Na1—H42A	136.9 (11)
O19—C17—C12	119.3 (6)	O28^i^—Na1—H42A	102.2 (18)
O18—C17—C12	115.6 (6)	Eu1—Na1—H42A	114 (2)
C17—O18—Eu1	125.3 (4)	Eu1^i^—Na1—H42A	119.1 (8)
C17—O18—Na1	130.5 (4)	H41A—Na1—H42A	91 (3)
Eu1—O18—Na1	100.66 (17)	Na1—O41—H41B	101 (7)
C13—C20—H20A	109.5	H41A—O41—H41B	99 (8)
C13—C20—H20B	109.5	Na1—O42—H42B	126 (7)
H20A—C20—H20B	109.5	H42A—O42—H42B	86 (10)
C13—C20—H20C	109.5	H43A—O43—H43B	93 (10)

Symmetry codes: (i) −*x*+3/2, *y*−1/2, −*z*+1/2; (ii) −*x*+3/2, *y*+1/2, −*z*+1/2.

### Hydrogen-bond geometry (Å, °)

**Table:**

*D*—H···*A*	*D*—H	H···*A*	*D*···*A*	*D*—H···*A*
O41—H41A···O29^i^	0.82 (2)	2.08 (6)	2.767 (7)	141 (8)
O41—H41B···O19	0.81 (2)	2.02 (3)	2.809 (8)	164 (9)
O42—H42A···O9	0.81 (4)	2.41 (10)	2.774 (9)	108 (8)
O42—H42B···O43	0.82 (4)	2.08 (4)	2.852 (10)	158 (10)
O43—H43A···O41^iii^	0.82 (3)	2.02 (6)	2.769 (9)	152 (11)
O43—H43B···O29^i^	0.83 (2)	2.14 (6)	2.884 (8)	149 (10)

Symmetry codes: (i) −*x*+3/2, *y*−1/2, −*z*+1/2; (iii) −*x*+2, −*y*, −*z*.
